# Olaparib Attenuates Demyelination and Neuroinflammation in an Organotypic Slice Culture Model of Metachromatic Leukodystrophy

**DOI:** 10.1007/s13311-023-01409-w

**Published:** 2023-07-31

**Authors:** Marianna Mekhaeil, Melissa Jane Conroy, Kumlesh Kumar Dev

**Affiliations:** 1 Drug Development Research Group, Department of Physiology, School of Medicine, Trinity College Dublin, Dublin, Dublin 2 Ireland; 2 Cancer Immunology Research Group, Department of Physiology, School of Medicine, Trinity College Dublin, Dublin, Dublin 2 Ireland

**Keywords:** Cerebellum, PARP-1, Demyelination, Neuro-inflammation, Organotypic slice cultures, Olaparib

## Abstract

Metachromatic leukodystrophy (MLD) is a severe demyelinating, autosomal recessive genetic leukodystrophy. The disease is underpinned by mutations in the arylsulfatase A gene (ARSA), resulting in deficient activity of the arylsulfatase A lysosomal enzyme and consequential accumulation of galactosylceramide-3-O-sulfate (sulfatide) in the brain. Using an ex vivo murine-derived organotypic cerebellar slice culture model, we demonstrate that sulfatide induces demyelination in a concentration-dependent manner. Interestingly, our novel data demonstrate that sulfatide-induced demyelination is underpinned by PARP-1 activation, oligodendrocyte loss, pro-inflammatory cytokine expression, astrogliosis, and microgliosis. Moreover, such sulfatide-induced effects can be attenuated by the treatment with the poly (ADP-ribose) polymerase 1 (PARP-1) inhibitor Olaparib (IC50∼100 nM) suggesting that this small molecule may be neuroprotective and limit toxin-induced demyelination. Our data support the idea that sulfatide is a key driver of demyelination and neuroinflammation in MLD and suggest that PARP-1 inhibitors have therapeutic utility in the sphere of rare demyelinating disease.

## Introduction

Metachromatic leukodystrophy (MLD) is a lysosomal disorder caused by recessive mutations in the arylsulfatase A gene (ARSA), encoding arylsulfatase A enzyme (ASA) [1]. ASA is essential for galactosylceramide-3-O-sulfate (sulfatide) metabolism [1]. Its deficiency results in the accumulation of sulfatides into lysosomal storage deposits in the central and peripheral nervous system. Sulfatides are the most abundant sphingolipids in myelin, accounting for 4% of its composition [1]. They have important functions in the maintenance of myelin [6]. In MLD, sulfatides accumulate in the oligodendrocytes, Schwann cells, phagocytes, astrocytes, and neurons, leading to demyelination [2]. Such sulfatide accumulation has been implicated as a key contributor to neuroinflammation and oxidative stress in MLD [3]. MLD is associated with the loss of muscle, cognitive function, and progressive loss of vision, with an estimated birth prevalence of approximately 1–2/100,000, and an incidence of 1/40,000 births [4]. This devastating demyelinating disease can be classified in a late-infantile, juvenile, and adult-onset type depending on the age of onset. All forms are characterized by a variety of neurological symptoms, which eventually lead to death if untreated. There is no curative treatment for MLD and novel therapeutic options are urgently required [4].

Poly (ADP-ribose) polymerase 1 (PARP-1) is the most abundant and well-characterized member of the PARP nuclear enzyme superfamily that catalyses the transfer of ADP-ribose units (PAR) from nicotinamide adenine dinucleotide (NAD +) to a broad panel of acceptor proteins such as histones and transcription factors [5]. PARP-1 is involved in a wide range of cellular processes including DNA repair, transcriptional regulation, and mitochondrial function [5]. An overactivation of PARP-1 has been associated with the pathogenesis of several brain disorders, such as Parkinson’s disease (PD), traumatic brain injury, and multiple sclerosis (MS) [6, 7]. PARP-1 overactivation causes excessive PAR synthesis, NAD + and ATP depletion, and ultimately, cell death [8]. In addition, PARP-1 plays a role in disease via co-activation of nuclear factor kappa-light-chain-enhancer of activated B cells (NF-κB), which induces the transcription of genes encoding proteins involved in inflammation, oxidation, and cell adhesion, such as tumor necrosis factor-α (TNF-α), inducible nitric oxide synthase (iNOS), and intercellular adhesion molecule-1 (ICAM-1) [8, 9]. Together these molecules promote inflammation, which in turn augments the expression of reactive oxygen species (ROS) and increases genomic instability as well as the sensitivity of surrounding cells to oxidation [10]. Considering the evidence of augmented DNA fragmentation, oxidative stress, inflammation, mitochondrial dysfunction, and BBB disruption in MLD pathology, PARP-1 could be a key mediator of neurodegeneration in MLD. Moreover, PARP-1 gene deletion or pharmacological inhibition could exert a neuroprotective effect in the setting of MLD.

Olaparib is a PARP-1 inhibitor which has been FDA-approved for the treatment of cancer. Interestingly, this small molecule inhibitor has shown efficacy in reducing oxidative stress, decreasing PAR synthesis, preventing NAD + depletion and cell death, and decreasing inflammation in neurological disease [6, 11]. Here, we have generated a novel mouse organotypic slice culture model of sulfatide-induced demyelination in which to uncover the role of PARP-1 in demyelination and neuro-inflammation and examine the therapeutic utility of Olaparib in MLD.

## Materials and Methods

### Pharmacological Compounds

Sulfatide (Sigma; 383906-24-9) comprises the major glycolipid components of myelin and was prepared as a 10 mM stock solution dissolved in 90% dimethyl sulfoxide (DMSO, Sigma; D8418). Olaparib (Bioscience; AZD2281) is a PARP-1 inhibitor (IC50 = 13 nM) [12] with partial binding affinity for PARP-2 that was prepared as a 20 mM stock solution in 90% DMSO.

### Generation of a Mouse Organotypic Cerebellar Slice Culture Model of Sulfatide-Induced Demyelination

Mouse organotypic cerebellar slice (OCS) cultures were generated from postnatal day 10 C57BL/6 mice (P10) provided by BioResources Unit, Trinity College Dublin (Ireland), as described previously [13]. All tissue was isolated in accordance with EU regulations and internal protocols approved by Trinity College Dublin ethical committee. Mice were sacrificed by decapitation, the skull removed, and the cerebellum separated from hindbrain. Cerebellum was cut into 400 μm parasagittal sections using a McIlwan tissue chopper. Tissue was placed into Opti-MEM (#31,985, Gibco, Thermo Fisher Scientific) and separated into individual slices under a dissection microscope. Five slices were placed per cell culture insert (PICMORG50, Merk Millipore, Burlington, MA, USA) and grown at 35.5 °C, 95% humidity, and 5% CO_2_. Slices were grown for the first 4 days in media containing 50% Opti-MEM, 25% Hank’s buffered salt solution (HBSS; #14,025–050, Gibco, Thermo Fisher Scientific), and 25% heat-inactivated horse serum (#26,050–088, Gibco, Thermo Fisher Scientific) supplemented with 2 mM Glutamax (#35,050, Gibco, Thermo Fisher Scientific), 28 mM D-Glucose (G8769, SigmaAldrich), 1% penicillin/streptomycin (pen/strep, Sigma; P4333) and HEPES (#15,630–056, Gibco, Thermo Fisher Scientific), At day 4, media was changed to a serum-free media containing 96% Neurobasal-A (#10,888–022, Gibco, Thermo Fisher Scientific) and 2% B-27 supplement (#17,504–044, Gibco, Thermo Fisher Scientific) supplemented with 1% penicillin/streptomycin (pen/strep, Sigma; P4333), 28 mM D-Glucose, 2 mM Glutamax and 10 mM HEPES. Media was changed again on day 10. To induce demyelination in the model, slices were treated with sulfatides (10 µM, 20 µM, 50 µM, 100 µM) at day 12 for 24 h.

### Elucidating the Rescuing Effects of Olaparib in a Mouse OCS Culture Model of Sulfatide-Induced Demyelination

OCS model of sulfatide-induced demyelination were generated as described in 2.2. To test the therapeutic utility of Olaparib in sulfatide-induced demyelinating disease at day 12, OCSs were exposed to sulfatide in the presence or absence of Olaparib (100 nM) for 24 h.

### Immunofluorescence of Slice Cultures

After sulfatide ± Olaparib treatments, the OCS cultures were fixed in 4% paraformaldehyde (PFA) for 7 min. Slices were washed with phosphate-buffered saline (PBS) twice for 10 min. Blocking and permeabilization were performed overnight at 4 °C in PBS containing 10% bovine serum albumin (BSA, Sigma) + 0.5% Triton X-100. Primary antibodies were diluted in 2% BSA in PBS + 0.1% Triton X-100 and incubated for 48 h at 4 °C. Slices were washed with PBS for 5 min and PBS + 0.1% Triton X-100 buffer three times for 10 min. Incubation with secondary antibodies was performed at 4 °C for 18 h. Slices were washed again, stained with Hoechst nuclear stain (diluted 1:10,000 in PBS) (Invitrogen; H21486), and mounted on microscope slides using ProLong^®^ Gold antifade reagent (ThermoFisher Scientific, P36934). Samples were stored at 4 °C in the dark until imaging was performed. Images were captured with a confocal microscope (Leica SP8) in 1024 × 1024 resolution at 200 frames per second (fps).

Primary antibodies included: rabbit anti-myelin basic protein (MBP) (Abcam, ab40390; 1/1,000 dilution), mouse monoclonal anti-myelin oligodendrocyte glycoprotein (MOG) (Millipore, MAB5680; 1/1,000 dilution), chicken anti-neurofilament heavy (NFH) (Millipore, AB5539; 1/1,000 dilution), mouse monoclonal anti-vimentin (Santa Cruz, sc-373717; 1/1,000 dilution), rabbit anti-ionized calcium-binding adapter molecule 1 (Iba1) (Wako, 019-19741; 1/1,000 dilution), mouse monoclonal anti-SMI-32 (Millipore, NE1023; 1/1,000 dilution), chicken anti-glial fibrillary acidic protein (GFAP) (Abcam, ab4674; 1/1,000 dilution), rabbit anti-Olig2 (Abcam, ab109186; 1/500 dilution), and mouse monoclonal anti-PARP-1 (Santa Cruz Biotech; sc-8007; 1/300 dilution). Secondary antibodies used included: goat anti-rabbit Alexa Fluor 488 (ThermoFisher Scientific, A11008; 1/1,000 dilution), goat anti-mouse Alexa fluor 488 (Invitrogen, A11001; 1/1,000 dilution), and goat anti-chicken IgY Alexa Fluor 633 (ThermoFisher Scientific, A21103; 1/1,000 dilution).

### Microscopy and Image Analysis of Slice Cultures

Immunofluorescence images of OCS cultures were captured at × 20 or × 40 magnification using a Leica SP8 confocal microscope. For each experiment (n = 5), there were five slices per treatment group and 5–6 fluorescence images were captured per slice. The areas of the cerebellum captured were kept consistent between treatment groups and cover most of the total area of the cerebellum. The images were exported as 8-bit.tif files for analysis using the software package FIJI (ImageJ, NIH, version 2.0.0). Intensity values were normalized to the average of control for each experiment and each marker. To analyze the expression of SMI-32 in the white matter tracts, a region of interest (ROI) containing predominantly white matter tracts was manually selected in each image and the fluorescence intensity was calculated. The proportion of this white matter area that stained positive for SMI-32 immunoreactivity (termed SMI-32 surface area) was quantified. For PARP-1, the fluorescence images were processed as follows: ROIs of cellular nuclei automatically generated by ImageJ using the Hoechst field were utilized to quantify the average gray value of the PARP-1 field. For cell count and surface area analysis, FIJI’s particle analyzer tool was employed. Briefly, images were converted from 8-bit to binary and a value of 20 pixels was set as the minimum particle size. A mask of the particles detected was generated to check the accuracy of the detection method. A numbered list of particles displaying the fluorescence intensity and the area of each were analyzed. For astrocyte morphology, FIJI’s skeletonized and AnalyzeSkeleton (2D/3D) software plugins were used. The outputs of these plugins summarize cell morphology in terms of astrocyte number of branches.

### Western Blot

After sulfatide ± Olaparib treatments, OCS were homogenized and sonicated in RIPA buffer containing protease inhibitors (cOmplete, Roche, 11697498001), and centrifuged at 14,000 rpm, and supernatant was collected. Samples were denatured and electrophoresis was performed on 12% SDS–polyacrylamide gels. Electrophoresis was followed by a semi-dry transfer to a PVDF membrane (Millipore, IPVH00010), which was then blocked in 5% BSA in PBS + 0.05% Tween for 1 h at room temperature. Incubation with primary antibody was performed overnight at 4 °C. Primary antibodies used were rabbit anti-MBP (Abcam, ab40390; 1/2,000 dilution), mouse monoclonal anti-MOG (Millipore, MAB5680; 1/2,000 dilution), mouse monoclonal anti-vimentin (Santa Cruz, sc-373717; 1/1,000 dilution), rabbit anti-Iba1 (Wako, 019-19741; 1/1,000 dilution), chicken anti-GFAP (Abcam, ab4674; 1/2,000 dilution), and rabbit anti-Olig2 (Abcam, ab109186; 1/1,500 dilution). The membranes were washed and incubated with secondary goat anti-rabbit (VWR, NA934; 1/5,000 dilution), donkey anti-chicken (Sigma, SAB4600127; 1/2,000 dilution), or goat anti-mouse (Thermo Fisher Scientific, A16066; 1/5,000 dilution) HRP-conjugated antibodies for 2 h at room temperature. Membranes were developed using a chemiluminescent HRP substrate (Millipore, WBKLS0500), and images were acquired on a C-Digit blot scanner with Image Studio 4.0 (LI-COR).

### Quantification of the Inflammatory Secretome by Enzyme-Linked Immunosorbent Assay (ELISA)

OCS were treated with sulfatides with or without Olaparib for 24 h. Media were then collected, and frozen at − 80 °C. Interleukin 6 (IL-6), tumor necrosis factor-alpha (TNF-α) interleukin 17A (IL-17A), macrophage inflammatory protein-1α (MIP-1α/CCL3), macrophage migration inhibitory factor (MIF), and interferon-gamma (IFN-γ) levels in OCS conditioned media were detected with mouse IL-6 ELISA kit according to the manufacturer’s instructions (R&D Systems; DY406), mouse TNF-α kit (R&D Systems; DY410), mouse IL-17A kit (ELISA Genie; MOFI01286), mouse CCL3 (R&D Systems; DY450), mouse MIF (R&D Systems; DY1978), and mouse IFN-γ (ELISA Genie; MOFI00047). Quantification of secreted IL-6, TNF-α, CCL3, and MIF in conditioned media was performed as per R&D Systems kit instructions. Briefly, 96-well ELISA plates (Thermo Scientific; 95029780) were coated overnight at 4 °C with capture antibodies diluted in Dulbecco's PBS (dPBS, Sigma; 14,190-094). The plates were washed three times with wash buffer (0.05% Tween 20 (Sigma; P7949), 10X PBS, pH 7.4) and then blocked for 2 h at room temperature with the appropriate reagent diluent. The plates were then washed three times with wash buffer, and any remaining buffer was removed from the wells by aspiration. A standard curve was prepared using serial dilutions of the recombinant protein diluted in the appropriate reagent diluents. The samples and standards were then incubated in the antibody-coated ELISA plate for 2 h at room temperature. The plate was then washed three times with wash buffer, and a detection antibody (diluted in reagent diluent) was added to each well for 2 h. Following three more washes, streptavidin-HRP diluted in reagent diluents was added to each well and incubated for 20 min at room temperature, protected from light. After an additional three washes, the wells were incubated with substrate solution (R&D systems; DY999) for 20 min at room temperature protected from light. The color reaction was stopped with the addition of 1 M H2SO4, and absorbance was read immediately using a plate reader at 450 nm (Labsystem Multiskan). For IL-17A and IFN-γ, pre-coated plates were washed three times before incubating with standards and samples for 2 h at room temperature according to manufacturer instructions. The standard curve was calculated by plotting the standards against the absorbance values, and the cytokine levels were measured in pg/ml.

### Statistical Analysis

All data were analyzed using GraphPad Prism 5 Software package (GraphPad Software, Inc.). The normality of the data was determined using the Shapiro-Wilk test. Where appropriate, histologic and biochemical data were analyzed using the parametric one or two-way ANOVA with the Tukey post-hoc test for multiple comparisons to assess significant differences between the values obtained for vehicle control, sulfatide-treated and Olaparib-treated organotypic slice cultures. For Western blot Newman–Keuls multiple comparisons post hoc tests were run in conjunction with one‐way ANOVAs and all groups were compared with one another. Mean fluorescence intensity as measured with ImageJ software was used as an arbitrary unit of measure. Raw data sets were normalized and presented as percentages of the control group average. The differences were considered significant if p < 0.05 and all values were expressed as the mean “standard error of the mean” (SEM). Where indicated, ‘n’ stands for the number of independent organotypic slice culture preparations performed on different experimental days. For each experiment, there were five technical replicates per treatment group and each experiment was repeated n = 3 or 4 or 5 times, as indicated in the appropriate figure legend. Separate experiments were counted as slices that were extracted from different brain tissue from different mouse litters on different experimental days. For each slice culture, 25 healthy slices were obtained from the cerebellum of five mouse littermates and were randomly separated into five different treatment groups. Therefore, if an experiment was repeated n = 5, a total of 25 cerebellar slices were stained and imaged.

## Results

### Sulfatide Induces Demyelination in Mouse OCS

**Fig. 1:**
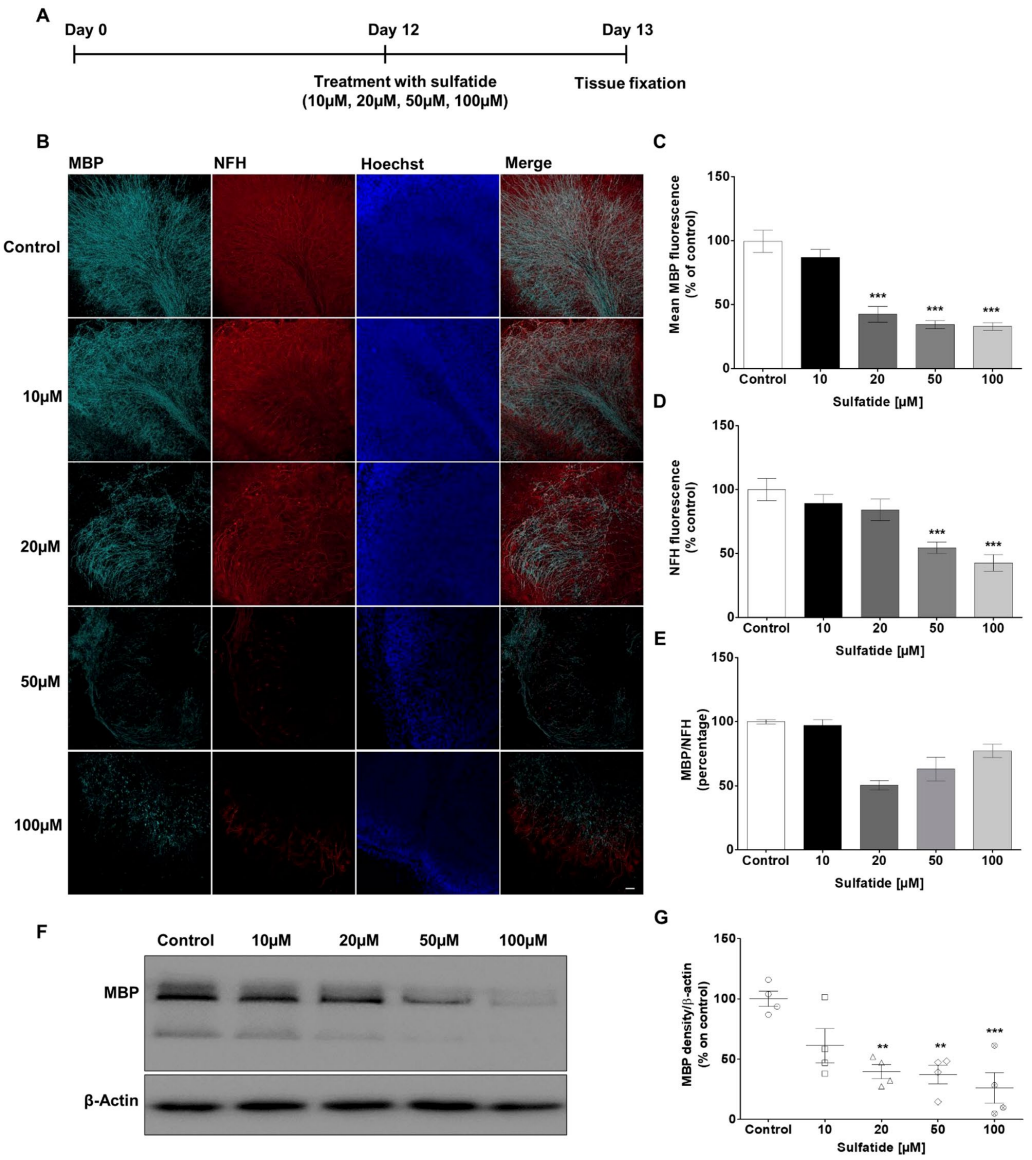
**Sulfatide induces demyelination in cerebellar slice cultures in a concentration-dependent manner.** OCS were treated with sulfatides (10 μM, 20 μM, 50 μM, 100 μM) for 24 h. **A** Schematic diagram explaining the experimental protocol for pharmacological treatments of ex vivo brain slice cultures. Cerebellar slices were cultured for 12 days and then treated for 24 h with sulfatides (10 μM, 20 μM, 50 μM, 100 μM) to trigger demyelination. **B** Representative confocal images displaying MBP (blue), NFH (red), and Hoechst (blue) immunostaining under treatment conditions indicated. Confocal images were captured at × 20 magnification. **C** Bar graph illustrating changes in the intensity of MBP fluorescence after the treatment with sulfatides. Quantification of confocal images shows a significant decrease in MBP fluorescence at 20 μM, 50 μM, and 100 μM sulfatide. **D** Bar graph illustrating changes in the intensity of NFH fluorescence after the treatment with sulfatides. Quantification of confocal images shows a significant decrease in NFH fluorescence with 50 μM and 100 μM sulfatide treatment. **E** Bar graph illustrating the ratio of MBP on NFH. Data are expressed as a percentage of control and presented ± SEM compared with control (n = 5). Statistical significance was determined by One-way ANOVA followed by Tukey multiple comparison test, ***p < 0.001. A number of 100 images were analyzed per condition. Scale bar, 100 µm. **F**–**G** Changes in the levels of MBP were quantified using Western blotting. Myelination as measured by MBP was significantly decreased by 20 μM, 50 μM, and 100 μM sulfatide. Data are expressed as a percentage of control and presented ± SEM compared with control (n = 4). Statistical significance was determined by One-way ANOVA followed by Newman–Keuls post-hoc test, **p < 0.01, ***p < 0.001

To effectively study the role of PARP-1 in MLD, a murine OCS model of sulfatide-induced demyelination was generated to recapitulate the disease setting ex vivo. OCS were exposed to increasing concentrations of sulfatides (10 µM, 20 µM, 50 µM, and 100 µM) for 24 h (Fig. 1A). Sulfatides induced demyelination in a concentration-dependent manner, as observed by expression of myelin basic protein (MBP) (10 µM: 86.7%; 20 µM: 42.3%, ***p < 0.001; 50 µM: 34.3%, ***p < 0.001; and 100 µM: 32.9%, ***p < 0.001, compared with control) (Fig. 1B and C). Importantly, the exposure of the slice cultures to sulfatide also decreased the expression of neurofilament H (NFH) (10 µM: 89.2%; 20 µM: 84.0%; 50 µM: 54.5%, ***p < 0.001; and 100 µM: 42.6%, ***p < 0.001, compared with control) (Fig. 1D). The ratio of MBP on NFH showed that demyelination at 20 μM sulfatides could be rescuable given that neuronal degeneration hasn’t occurred yet (Fig. 1E). Taken together, these results demonstrate that sulfatide induces demyelination and neuronal toxicity in OCS cultures. A concentration of 20 μM sulfatides was selected for subsequent experiments.

Western blotting analysis confirmed that high concentrations of sulfatide disrupt myelination. This was shown by the total expression levels of MBP in homogenized cerebellum tissue. OCS were exposed to increasing concentrations of sulfatides (10 µM, 20 µM, 50 µM, and 100 µM) for 24 h, and protein levels of MBP with respect to total β-actin expression were measured (Fig. 1F). Sulfatide-induced decreases in MBP levels were concentration-dependent (10 µM: 71.6%; 20 µM: 46.9%, **p < 0.01; 50 µM: 43.6%, **p < 0.01; and 100 µM: 30.5%, ***p < 0.001, compared with control) (Fig. 1G).

### Sulfatide-Induced Demyelination is Mediated by PARP-1 Overexpression in Mouse OCS

**Fig. 2:**
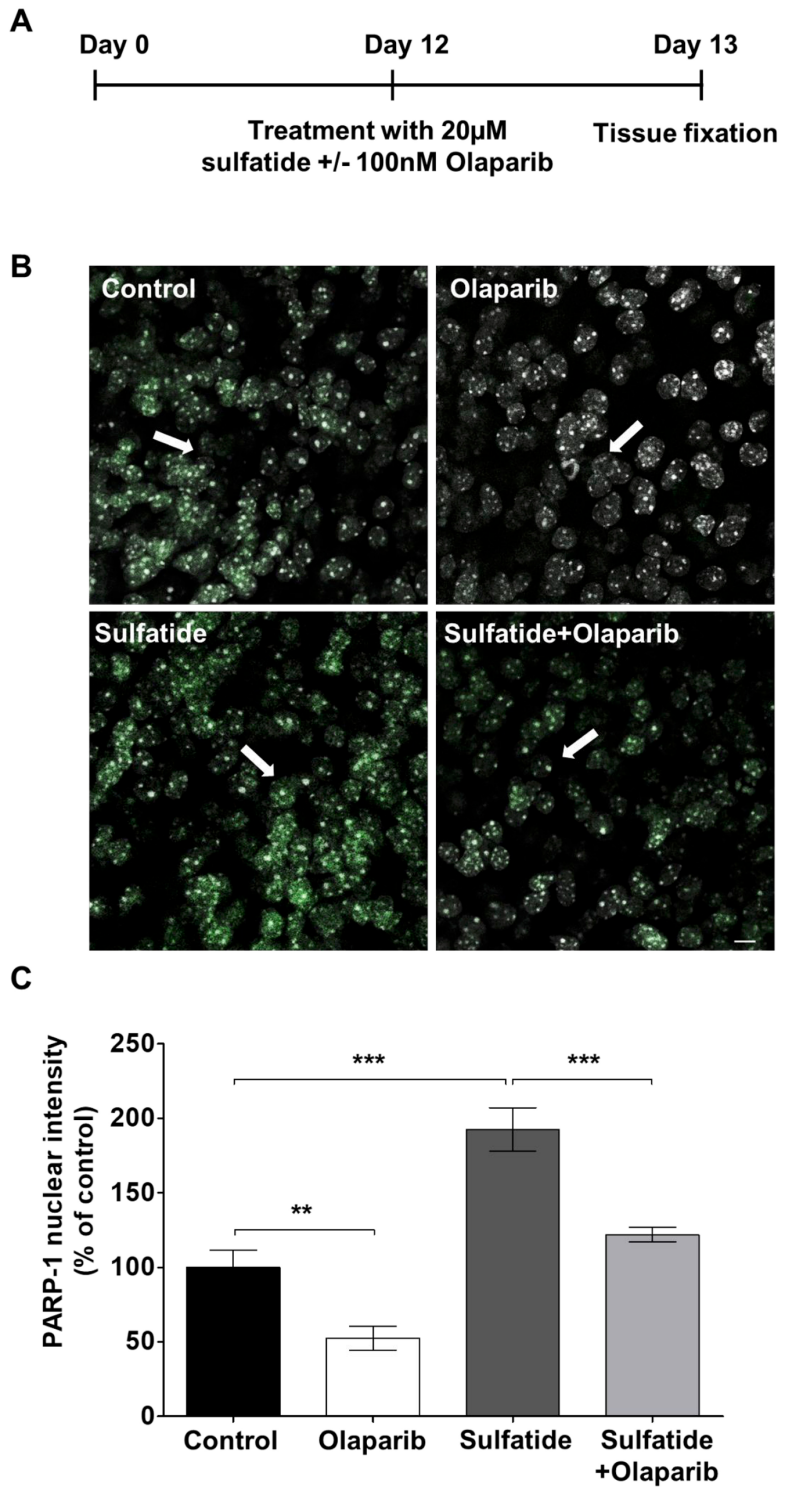
**Sulfatide-induced demyelination is mediated by PARP-1 expression in slice cultures. A** Schematic diagram explaining the experimental protocol for pharmacological treatments of ex vivo brain slice cultures. Cerebellar slices were cultured for 12 days and then treated for 24 h with 20 μM sulfatides in the presence or absence of 100 nM Olaparib. **B** Representative confocal images displaying PARP-1 (green) and Hoechst (grey) immunostaining under treatment conditions indicated. Confocal images were captured at × 40 magnification. **C** Bar graphs showing that treatment with Olaparib (100 nM) reduced sulfatide-induced PARP-1 expression in nuclei (20 µM). A number of 100 images were analyzed per condition. Semi-quantitative analysis of PARP-1-associated fluorescence in the nuclei. Data are expressed as a percentage of control and presented ± SEM compared with control (n = 5). Statistical significance was determined by One-way ANOVA followed by Tukey multiple comparison test, **p < 0.01, ***p < 0.001. A number of 100 images were analyzed per condition. Scale bar, 100 µm

PARP-1 is involved in the pathogenesis of various neurodegenerative diseases [6] and is regulated during demyelination as well as remyelination [14]. To assess its role in MLD, the effects of sulfatides and Olaparib on PARP-1 activation in our OCS cultures were investigated by measuring the changes in PARP-1 expression in the nucleus (Fig. 2A). Olaparib alone significantly decreased PARP-1 expression in nuclei compared to control (52.4%, **p < 0.01) (Fig. 2B and C). Treatment of slice cultures with 20 μM sulfatides for 24 h induced PARP-1 expression in the nuclei of surviving cells compared to control (192.5%, ***p < 0.001) (Fig. 2B and C). Importantly, these changes in PARP-1 fluorescence were reduced by treatment with 100 nM Olaparib (Sulfatide: 192.5% vs Sulfatide + Olaparib: 121.9%, ***p < 0.001) (Fig. 2B and C). Overall, these results support the idea that sulfatide-induced toxicity is at least in part mediated by PARP-1 overactivation and that Olaparib holds utility to attenuate these effects.

### Olaparib Inhibits Sulfatide-Induced Demyelination in OCS

**Fig. 3:**
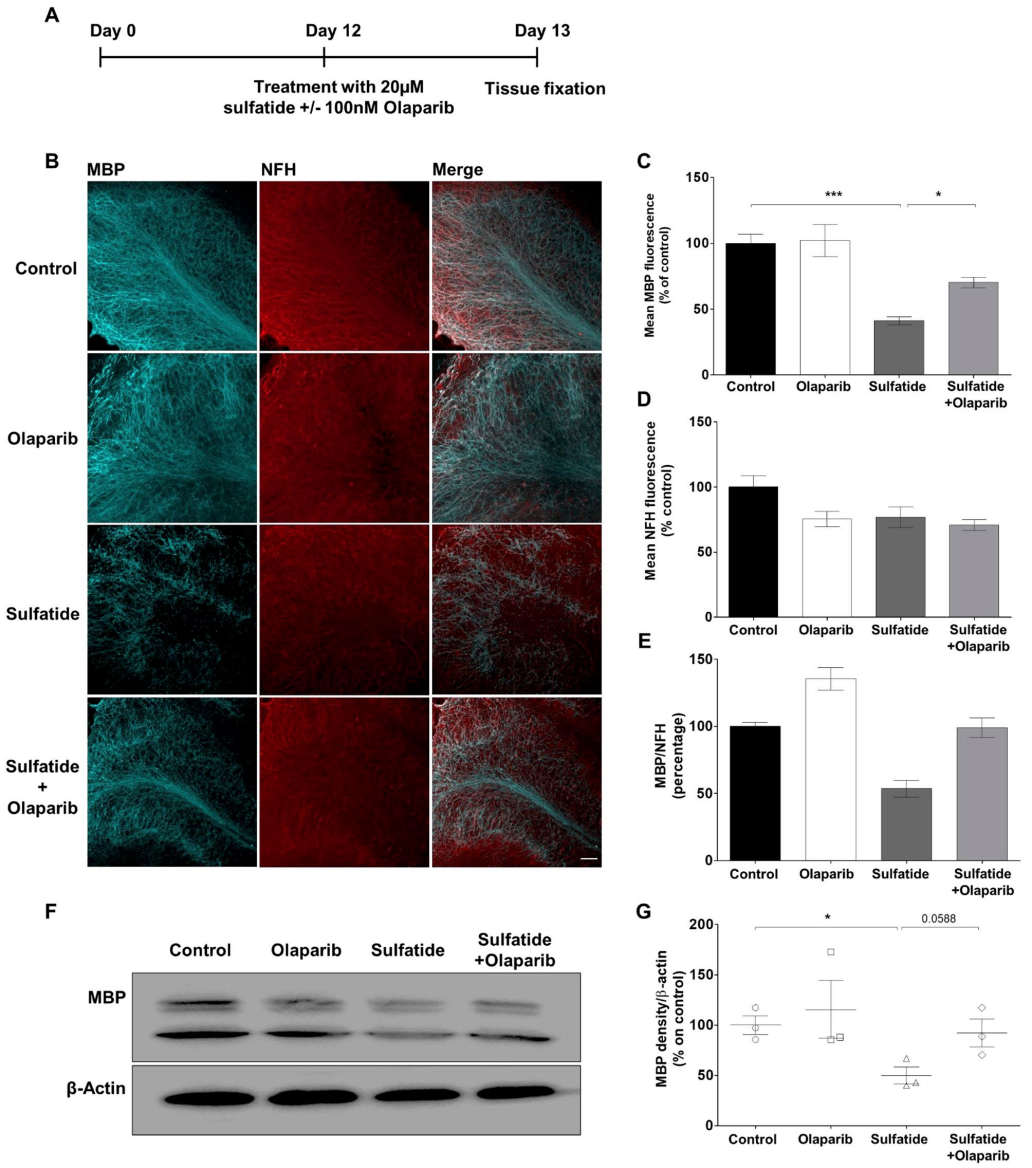
**Olaparib attenuates sulfatide-induced demyelination in cerebellar slice cultures. A** OCS slices were treated with sulfatides (20 µM) in the presence or absence of Olaparib (100 nM) for 24 h. **B** Representative confocal images displaying MBP (blue) and NFH (red) immunostaining under treatment conditions indicated. Confocal images were captured at × 20 magnification. **C** Bar graph illustrating changes in the intensity of MBP fluorescence after the treatment with sulfatides (20 µM) and with or without Olaparib (100 nM). Quantification of confocal images shows a significant decrease in MBP fluorescence, which is attenuated by Olaparib. **D** Bar graph illustrating changes in the intensity of NFH fluorescence after the treatment with sulfatides (20 µM) and with or without Olaparib (100 nM). Quantification of confocal images shows no significant decrease in NFH fluorescence with sulfatide and Olaparib treatment. **E** Bar graph illustrating the ratio of MBP on NFH. Data are expressed as a percentage of control and presented ± SEM compared with control (n = 5). Statistical significance was determined by One-way ANOVA followed by Tukey multiple comparison test, *p < 0.05, ***p < 0.001. A number of 100 images were analyzed per condition. Scale bar, 100 µm. **F–G** Total MBP protein levels were measured by western blotting. Bar graph illustrating reduced MBP protein levels following sulfatide exposure, which is attenuated by Olaparib. Data are expressed as a percentage of control and presented ± SEM compared with control (n = 3). Statistical significance was determined by One-way ANOVA followed by Newman–Keuls post-hoc test, *p < 0.05

**Fig. 4:**
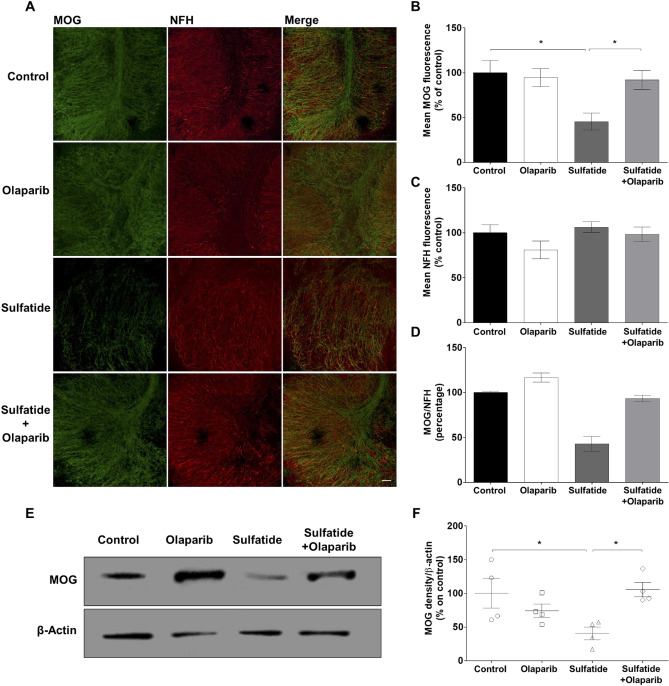
**Sulfatide-mediated reduction of MOG expression is attenuated by Olaparib.** OCS were treated with sulfatides (20 µM) in the presence or absence of Olaparib (100 nM) for 24 h. **A** Representative confocal images displaying MOG (green) and NFH (red) immunostaining under treatment conditions indicated. Confocal images were captured at × 20 magnification. **B** Bar graph illustrating changes in the intensity of MOG fluorescence after the treatment with sulfatides (20 µM) and with or without Olaparib (100 nM). Quantification of confocal images shows a significant decrease in MOG fluorescence, which is attenuated by Olaparib. **C** Bar graph illustrating changes in the intensity of NFH fluorescence after the treatment with sulfatides (20 µM) and with or without Olaparib (100 nM). Quantification of confocal images shows no significant decrease in NFH fluorescence with sulfatide and Olaparib treatment. **D** Bar graph illustrating the ratio of MOG on NFH. Data are expressed as a percentage of control and presented ± SEM compared with control (n = 5). Statistical significance was determined by One-way ANOVA followed by Tukey multiple comparison test, *p < 0.05. A number of 100 images were analyzed per condition. Scale bar, 100 µm. **E–F** Total MOG protein levels were measured by western blotting. Bar graph illustrating reduced MOG protein levels following sulfatide exposure, which is attenuated by Olaparib. Data are expressed as a percentage of control and presented ± SEM compared with control (n = 4). Statistical significance was determined by One-way ANOVA followed by Newman–Keuls post-hoc test, *p < 0.05

In the current study, the effect of Olaparib on sulfatide-induced demyelination was examined. OCS cultures were exposed to sulfatide (20 μM) in the presence or absence of Olaparib (100 nM) for 24 h (Fig. 3A). Olaparib alone elicited no significant effects on myelin and neuronal state compared to control, as determined by MBP and NFH immunostaining (Fig. 3B). More importantly, 20 μM sulfatide induced significant demyelination (41.21%, ***p < 0.001), as measured by the loss of MBP staining, which was significantly attenuated by Olaparib (Sulfatide: 41.21% vs Sulfatide + Olaparib: 70.19%, *p < 0.05) (Fig. 3C). Sulfatide induced a modest, although not significant, decrease in the levels of NFH (Fig. 3D). The ratio of MBP on NFH showed that demyelination at 20 μM sulfatides is in part rescued by Olaparib (Fig. 3E). The effect of 20 µM sulfatide on OCS treated in the presence or absence of 100 nM Olaparib was assessed on MBP protein levels (Fig. 3F). Levels of MBP were reduced in OCS treated with sulfatides (MBP: 49.7%, *p < 0.05; compared with control), and were increased by Olaparib (MBP: 92.0%, *p* = 0.0588; compared with control) (Fig. 3F and G). When examining the expression levels of myelin oligodendrocyte glycoprotein (MOG), similar results were found (Fig. 4A); 20 μM sulfatide treatment induced a significant reduction in the level of MOG (45.5%, *p < 0.05) compared to control, which was attenuated by treatment with 100 nM Olaparib (Sulfatide: 45.5% vs Sulfatide + Olaparib: 91.99%, *p < 0.05) (Fig. 4B). Again, changes in NFH fluorescence intensity were not significant (Fig. 4C and D). These data strongly suggest that Olaparib can limit demyelination caused by sulfatide accumulation. Total levels of MOG were also reduced in OCS treated with sulfatides (MOG: 40.59%, *p < 0.05 compared with control), and were increased by Olaparib (MOG: 105.4%, *p < 0.05 compared with control) (Fig. 4E and F). The patterns of total MOG and MBP expression mimic the immunofluorescence results, suggesting that PARP-1 is detrimental to myelin formation and that Olaparib may rescue sulfatide-induced demyelination.

### Effects of Olaparib on Oligodendrocytes in Cerebellar Slices Treated with Sulfatide

**Fig. 5:**
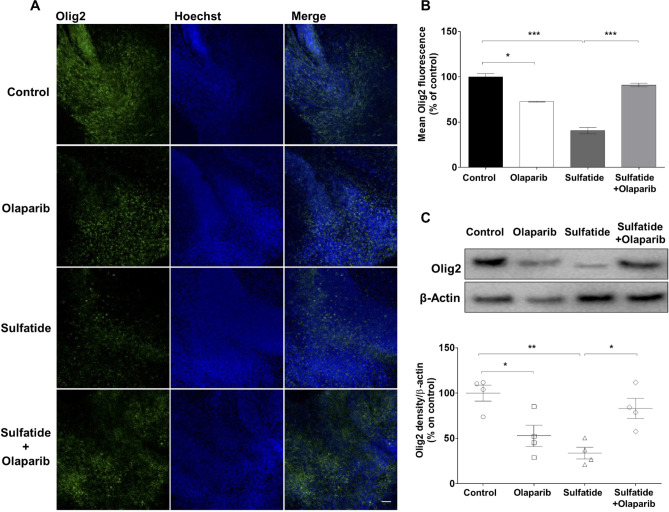
**Olaparib reduces sulfatide-induced oligodendrocyte death.** OCS were treated with sulfatides (20 µM) in the presence or absence of Olaparib (100 nM) for 24 h. **A** Representative confocal images displaying Olig2 staining under treatment conditions indicated. Confocal images were captured at × 20 magnification. **B** Bar graph illustrating that treatment with Olaparib alone and sulfatides significantly decreased Olig2 fluorescence compared with control. Importantly, sulfatide-induced decrease is attenuated with Olaparib. Data are expressed as a percentage of control and presented ± SEM compared with control (n = 5). Statistical significance was determined by One-way ANOVA followed by Tukey multiple comparison test, *p < 0.05, ***p < 0.001. A number of 100 images were analyzed per condition. Scale bar, 100 µm. **C** Changes in Olig2 expression levels were assessed by Western blotting. Sulfatide-induced Olig2 decrease was attenuated by Olaparib. Data are expressed as a percentage of control and presented ± SEM compared with control (n = 4). Statistical significance was determined by One-way ANOVA followed by Newman–Keuls post-hoc test, *p < 0.05, **p < 0.01

In MLD sulfatides mainly accumulate in oligodendrocytes, therefore we assessed the therapeutic effects of Olaparib in OCS treated with 20 µM sulfatide by staining with Olig2 (Fig. 5A). A significant change in Olig2 fluorescence was measured in cerebellar slices treated with 100 nM Olaparib alone (72.6%, *p < 0.05 compared with control) in accordance with previous studies [14] (Fig. 5B). Importantly, sulfatide induced a deeper decrease in Olig2 fluorescence (40.66%, ***p < 0.001 compared with control) and Olaparib significantly limited this decrease (90.5%, ***p < 0.001) (Fig. 5B). These results were confirmed by western blot which showed that sulfatide-induced decreases in total Olig2 levels were significantly attenuated by Olaparib (Olaparib: 52.8%, *p < 0.05; Sulfatide: 33.9%, **p < 0.01 compared with control; Sulfatide + Olaparib: 83.1%, *p < 0.05 compared with Sulfatide) (Fig. 5C).

### Olaparib Lessens Sulfatide-Induced Astrocytes Impairment in Cerebellar Slices

**Fig. 6:**
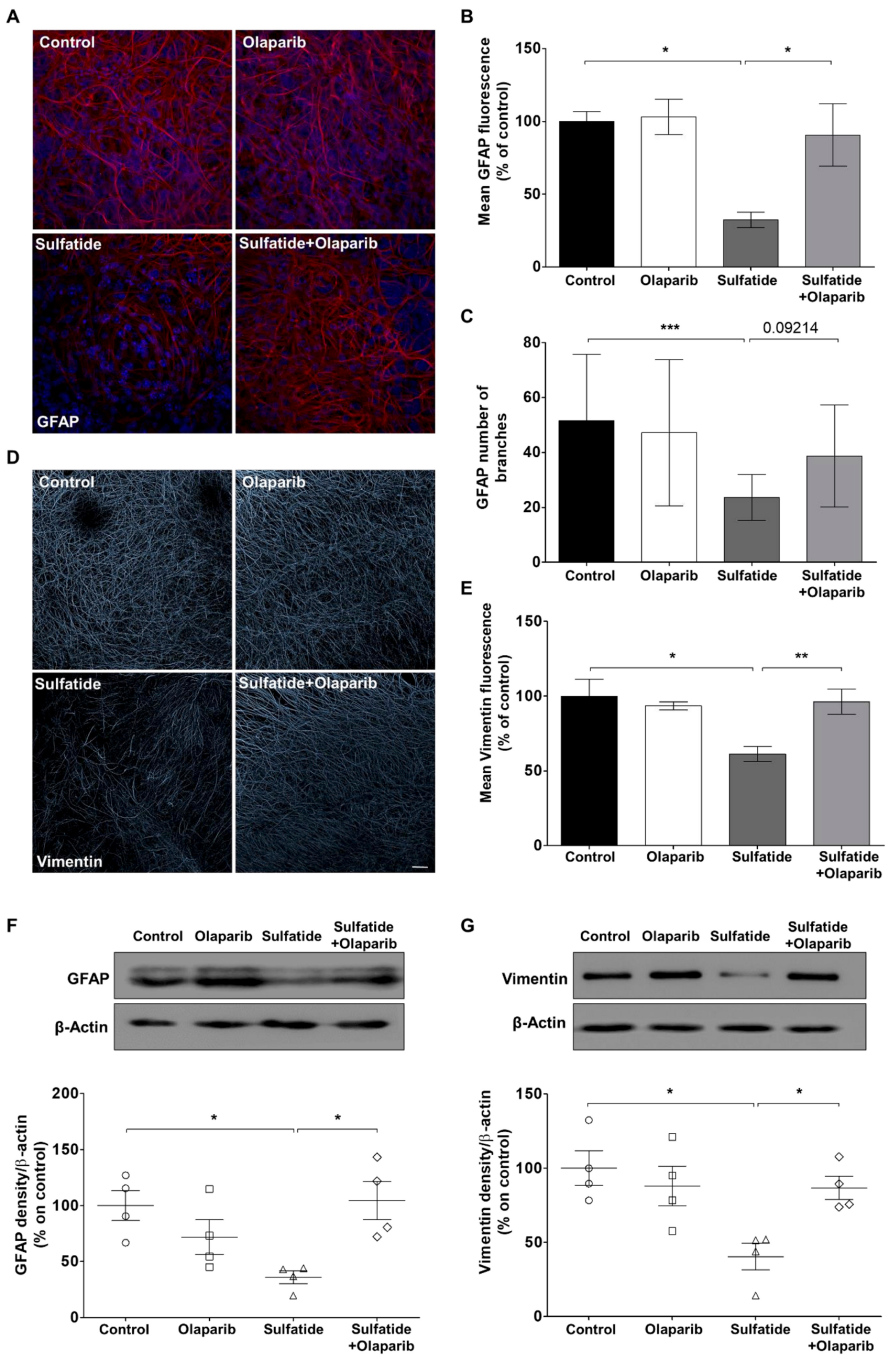
**Sulfatide-induced reductions in GFAP and vimentin in cerebellar slices is attenuated by Olaparib. A** Representative confocal images displaying GFAP staining under treatment conditions indicated. Confocal images were captured at × 40 magnification showing that treatment with 100 nM Olaparib attenuates astrocyte fluorescence intensity and number of branches decrease induced by 20 µM sulfatides. **B** Bar graph illustrating that treatment with sulfatides (20 µM) for 24 h significantly decreased GFAP fluorescence compared with control. Importantly, this decrease is attenuated with Olaparib (100 nM). **C** Bar graph illustrating that Olaparib attenuates sulfatide-induced decrease in GFAP number of branches compared with control. **D** Representative confocal images displaying vimentin staining under treatment conditions indicated. Confocal images were captured at × 20 magnification showing that treatment with 100 nM Olaparib attenuates vimentin fluorescence intensity decrease induced by 20 µM sulfatides. € Bar graph illustrating that treatment with sulfatides (20 µM) for 24 h significantly decreased vimentin fluorescence compared with control. Importantly, this decrease is attenuated with Olaparib (100 nM). Data are expressed as a percentage of control and presented ± SEM compared with control (n = 5). Statistical significance was determined by One-way ANOVA followed by Tukey multiple comparison test, *p < 0.05, ***p < 0.001, **p < 0.01. A number of 100 images were analyzed per condition. Scale bar, 100 µm. **F–G** Changes in GFAP and vimentin expression levels were assessed by Western blotting. Bar graph illustrating reduced GFAP (**F**) and vimentin (**G**) protein levels following sulfatide exposure, which is attenuated by Olaparib. Data are expressed as a percentage of control and presented ± SEM compared with control (n = 4). Statistical significance was determined by One-way ANOVA followed by Newman–Keuls post-hoc test, *p < 0.05

Astrocytes play an essential role in supporting myelination [15], participate in innate immune responses in the CNS, and have been implicated as drivers of neuroinflammation, which is a key driver of MLD disease [16]. Therefore, the effects of sulfatides (20 µM) and Olaparib (100 nM) on astrocytes activation in OCS were assessed using GFAP (mature astrocytes) and vimentin (neural progenitor cells/ immature astroglia), as astrocyte markers (Fig. 6A and D) [17]. A modest change in GFAP and vimentin fluorescence was observed in cerebellar slices treated with 100 nM Olaparib alone, however, this was not significantly different from control (GFAP: 103.09%; vimentin: 93.4% compared with control) (Fig. 6B and E). Importantly, sulfatide induced a decrease in GFAP fluorescence (32.3%, *p < 0.05 compared with control) and altered astrocyte morphology by reducing the number of branches (Control: 51 vs Sulfatide: 23, ***p < 0.001) (Fig. 6B and C). Co-treatment with Olaparib significantly limited sulfatide-mediated astrocyte toxicity by attenuating the decrease in the number of branches per astrocyte (Sulfatide: 23 vs Sulfatide + Olaparib: 38) and GFAP fluorescence intensity (32.3% vs 90.57%, *p < 0.05) (Fig. 6B and C). Sulfatide also induced a decrease in vimentin fluorescence (61.32%, *p < 0.05 compared with control) (Fig. 6D and E), and Olaparib significantly attenuated this (Sulfatide: 61.32% vs Sulfatide + Olaparib: 96.2%, **p < 0.01) (Fig. 6D and E). Total GFAP and vimentin levels were also decreased by sulfatide (GFAP: 36.8%, *p < 0.05; Vimentin 40.3%, *p < 0.05 compared with control), and increased by co-treatment with Olaparib (GFAP, Sulfatide: 36.8% vs Sulfatide + Olaparib: 104.4%, *p < 0.05; Vimentin, Sulfatide: 40.3% vs Sulfatide + Olaparib: 86.6%, *p < 0.05) (Fig. 6F and G). Overall, these data suggest that astrocytes have an altered function in sulfatide-treated cerebellum, and this is modulated by Olaparib.

### Olaparib Attenuates Sulfatide-Induced Microglia Activation

**Fig. 7:**
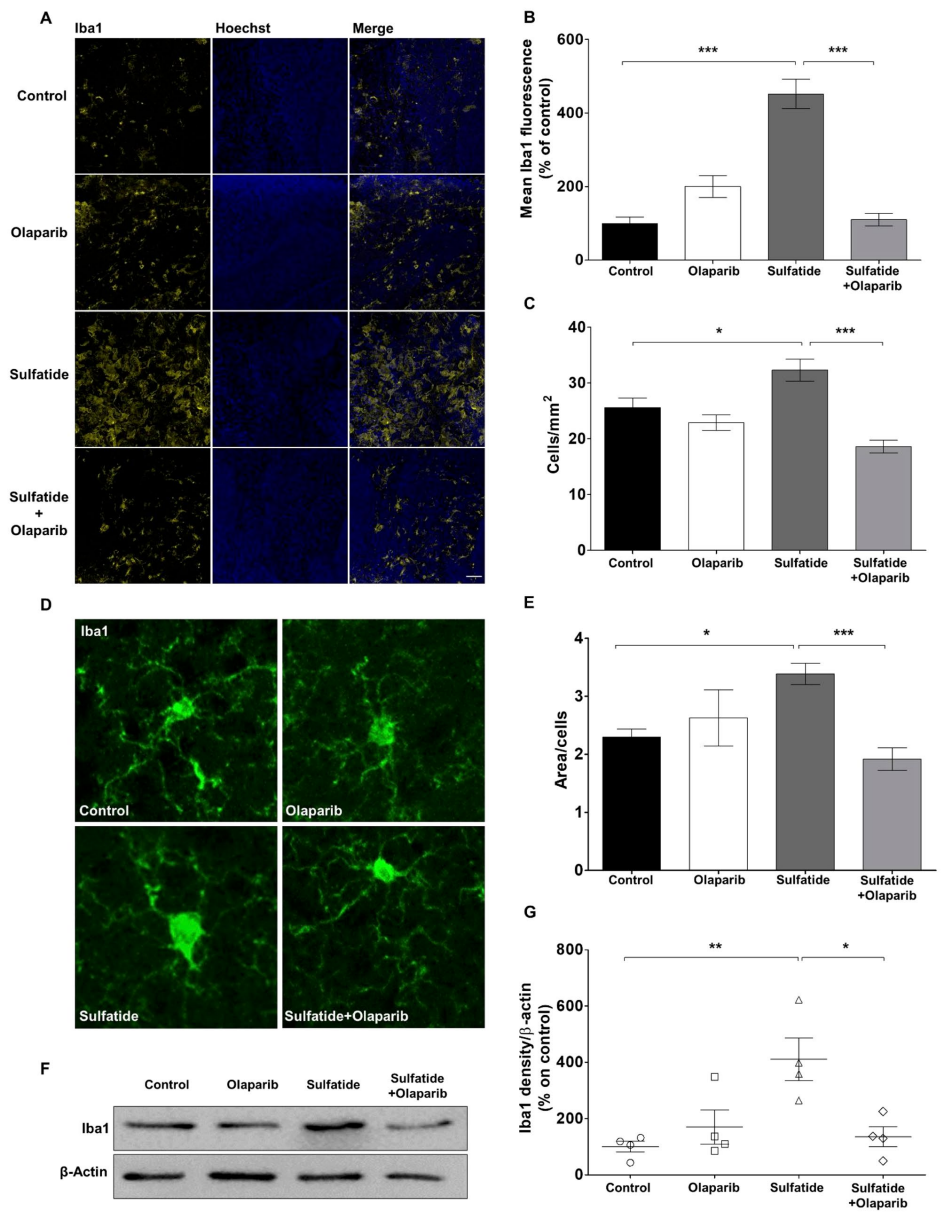
**Olaparib attenuates sulfatide-induced microglia reactivity.** OCS were treated with sulfatides (20 µM) in the presence or absence of Olaparib (100 nM) for 24 h. **A** Representative confocal images displaying Iba1 (yellow) and Hoechst (blue) immunostaining under treatment conditions indicated. Confocal images were captured at × 20 magnification **B** Bar graph illustrating changes in the intensity of Iba1 fluorescence after the treatment with sulfatides (20 µM) and with or without Olaparib (100 nM) for 24 h. Data are expressed as a percentage of control **C** Iba1 positive cells counting shows the sulfatides increase microglia proliferation and Olaparib reduces it. **D** Representative confocal images displaying Iba1 immunostaining under treatment conditions indicated. Confocal images were captured at × 40 magnification. **E** Quantification of cell surface on number of cells showed a significant increase in Iba1 surface in sulfatide-treated slices compared with control, which is attenuated by Olaparib. Data are expressed as a percentage of control and presented ± SEM compared with control (n = 5). Statistical significance was determined by One-way ANOVA followed by Tukey multiple comparison test, *p < 0.05, ***p < 0.001. A number of 100 images were analyzed per condition. Scale bar, 100 µm. (**F**–**G**) Changes in Iba1 expression levels were assessed by Western blotting. Bar graph illustrating increased Iba1 protein levels following sulfatide exposure, which is attenuated by Olaparib. Data are expressed as a percentage of control and presented ± SEM compared with control (n = 4). Statistical significance was determined by One-way ANOVA followed by Newman–Keuls post-hoc test, *p < 0.05, **p < 0.01

PARP-1 activation is involved in microglial activation, proliferation, and production of pro-inflammatory molecules [18]. Furthermore, PARP-1 deletion or inhibition in microglia promotes neuroprotection in the injured brain [19]. Hence, the effects of sulfatides and Olaparib on microglia were investigated. Treatment of slice cultures with 20 µM sulfatides induced a significant increase in microglia activation compared to control as shown by ionised calcium-binding adapter molecule 1 (Iba1) fluorescence (451.9%, ***p < 0.001 compared with control) (Fig. 7A and B). Importantly, these changes in Iba1 fluorescence were reduced by treatment with Olaparib (100 nM) (Sulfatide: 451.9% vs Sulfatide + Olaparib: 110.1, ***p < 0.001) (Fig. 7B). In addition, Olaparib reduced sulfatide-induced microglia proliferation as measured by Iba1 cell counting (Sulfatide: 32.3 vs Sulfatide + Olaparib: 18.5, ***p < 0.001) (Fig. 7C). Given the well-established association between microglia morphology and their function that classifies them as “resting” when they possess short, fine processes and are ramified, and “activated” when they appear spherical in shape and lack processes [20], the effects of sulfatides and Olaparib on microglia morphology were studied. The analysis of the total surface of Iba1 on the number of cells shows a significant increase when slices were treated with 20 µM sulfatides (Control: 2.29 vs Sulfatide: 3.38, *p < 0.05). Olaparib alone had no significant effect on Iba1 volume, while co-treatment of sulfatides and Olaparib significantly reduced microglial surface compared with sulfatides treatment alone indicating Olaparib’s ability to attenuate sulfatide-induced microglial activation (Sulfatide: 3.38 vs Sulfatide + Olaparib 1.92, ***p < 0.001) (Fig. 7D and E). Olaparib also attenuated sulfatide-induced increases in total Iba1 levels as shown by western blot (Sulfatide: 410.9%, **p < 0.01 compared with control; Sulfatide + Olaparib: 129.5%, *p < 0.05 compared with Sulfatide) (Fig. 7F and G). These observations demonstrate increased Iba1 expression and activation in sulfatide-treated OCS, which is attenuated significantly by Olaparib.

### Increased Cytokine and Chemokine Profile in Sulfatide-Treated Cerebellar Slices

**Fig. 8:**
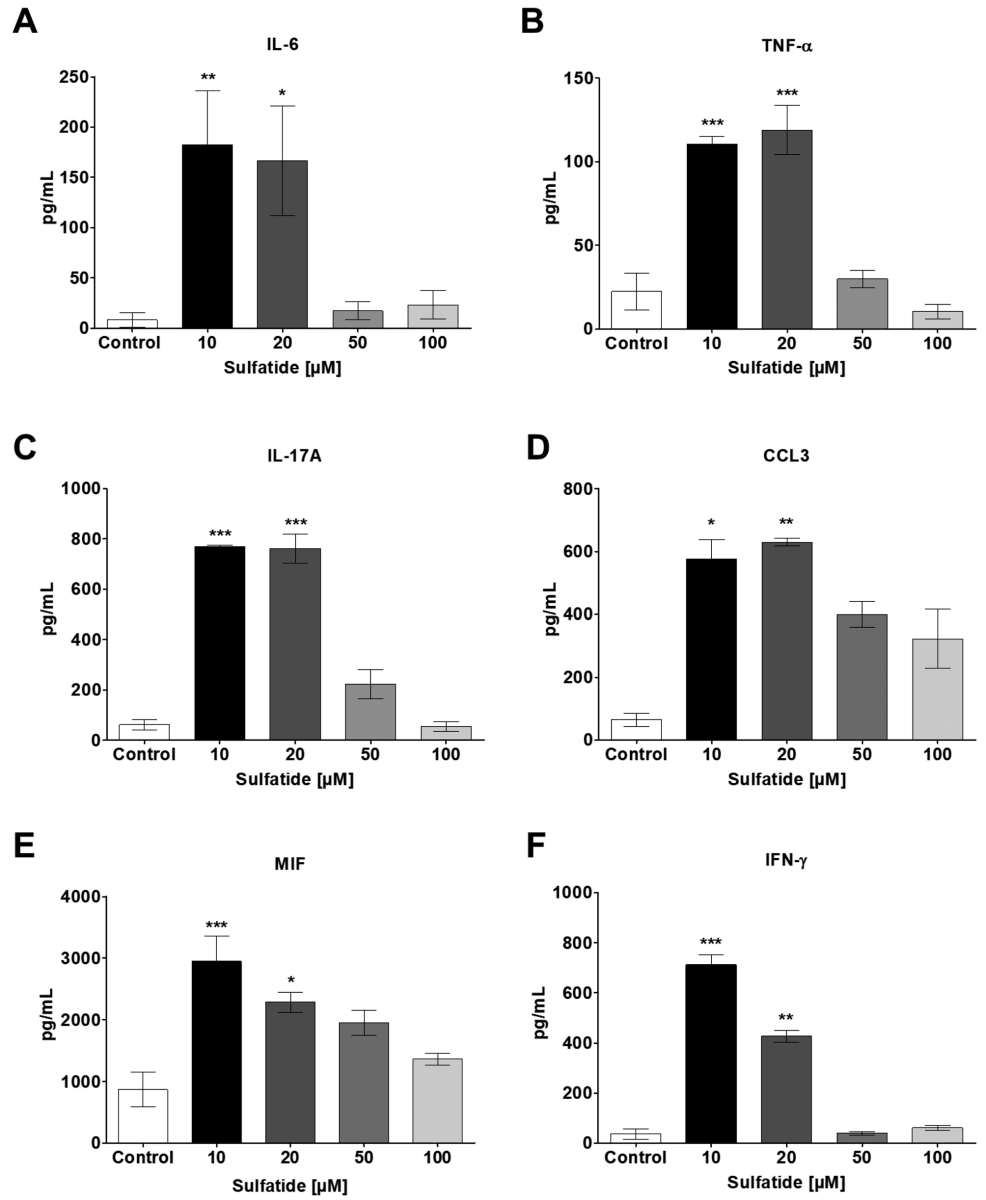
**Sulfatides increase cytokine and chemokine release from slice cultures.** Cytokine and chemokine release from control and sulfatide-stimulated slice cultures were measured after 24 h. Sulfatides (10 µM, 20 µM, 50 µM, and 100 µM) showed a significant increase in **A** IL-6, **B** TNF-α, **C** IL-17A, **D** CCL3, **E** MIF, and **F** IFN-γ release into the slice culture medium, as measured by ELISA. Data from the ELISA assays are presented as mean ± SEM. IL-6, TNF-α, IL-17A, CCL3, MIF, and IFN-γ ELISA's were repeated n = 5. Statistical analysis was performed using One-way ANOVA followed by Tukey’s post hoc test; *p < 0.05, **p < 0.01, ***p < 0.001

MLD patients exhibit augmented levels of pro-inflammatory cytokines and chemokines including CCL3, IL-1Ra, IL-8, and CCL4 in the cerebral spinal fluid (CSF) [16]. Since our data strongly indicate that sulfatides induce microglia activation, we next interrogated their effects on the secretion of pro-inflammatory cytokines and chemokines from OCS cultures. Treatment of cerebellar slices with sulfatides (10 μM, 20 μM, 50 μM, 100 μM) for 24 h resulted in increased soluble levels of IL-6, IL-17A, TNF-α, CCL3, MIF, and IFN-γ, which were significant at doses of 10 μM and 20 μM sulfatides, compared to the untreated control: IL-6 (Control: 8.2 pg/ml; 10 μM: 182.4 pg/ml, **p < 0.01; 20: μM 166.5 pg/ml, *p < 0.05; Fig. 8A), TNF-α (Control: 22.3 pg/ml; 10 μM: 110.4 pg/ml, ***p < 0.001; 20 μM: 118.9 pg/ml, ***p < 0.001; Fig. 8B), IL-17A (Control: 61.5 pg/ml; 10 μM: 769.4 pg/ml, ***p < 0.001; 20 μM: 762.2 pg/ml, ***p < 0.001; Fig. 8C), CCL3 (Control: 64.7 pg/ml; 10 μM: 572.2 pg/ml, *p < 0.05; 20 μM: 629.3 pg/ml, **p < 0.01; Fig. 8D), MIF (Control: 869.2 pg/ml; 10 μM: 2949.4 pg/ml, ***p < 0.001; 20 μM: 2287.0 pg/ml, *p < 0.05; Fig. 8E), and IFN-γ (Control: 36.6 pg/ml; 10 μM: 711.8 pg/ml, ***p < 0.001; 20 μM: 426.8 pg/ml, **p < 0.01; Fig. 8F). Taken together, these data suggest that exposure to sulfatides promotes inflammation in addition to the observed increases in demyelination and axonal damage.

### Olaparib Significantly Reduces Pro-Inflammatory Cytokine and Chemokine Release from Sulfatide-Treated Organotypic Slice Cultures

**Fig. 9:**
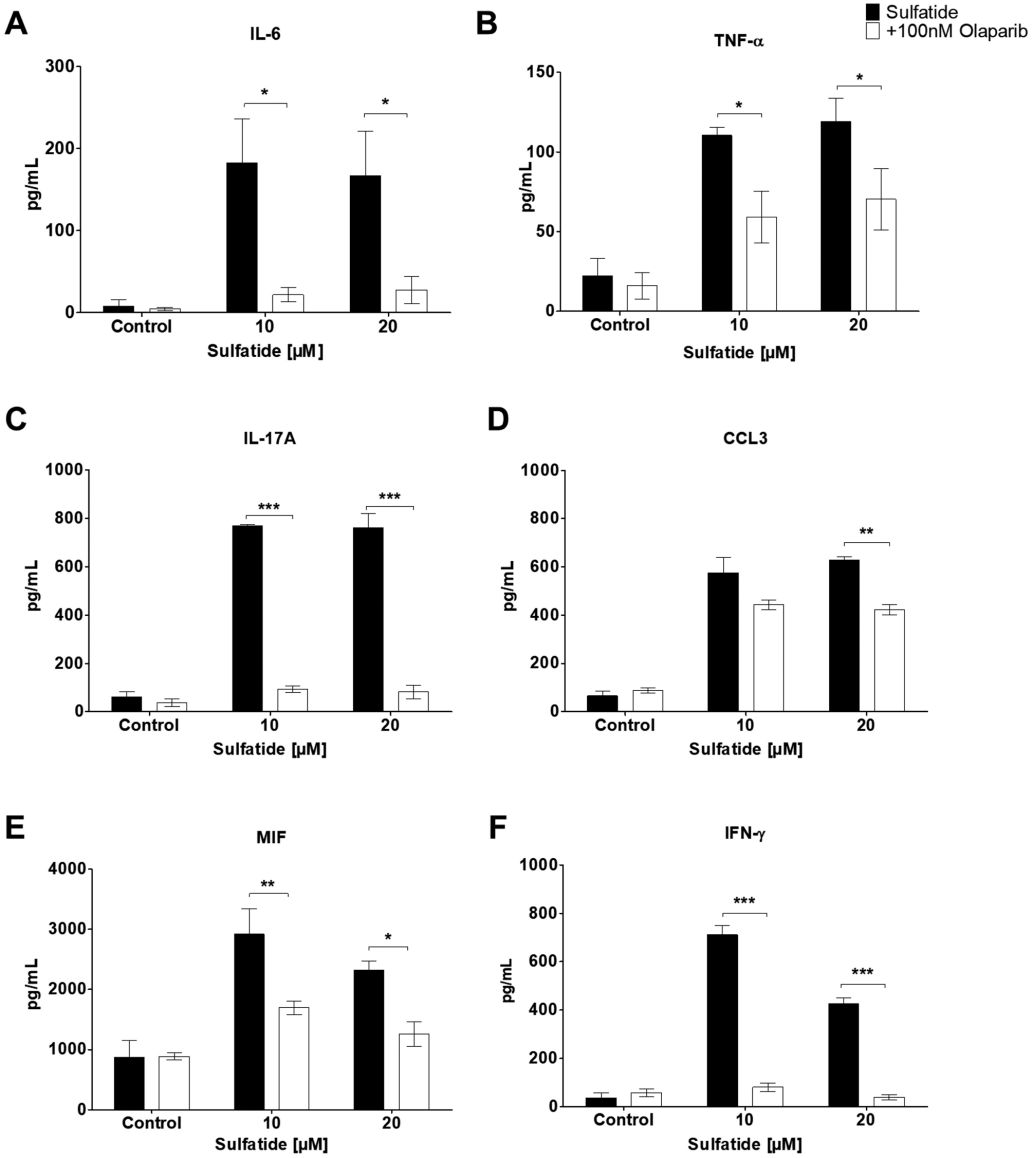
**PARP-1 modulates cytokine and chemokine release from sulfatide-treated organotypic slice cultures.** Cytokine and chemokine release from control and sulfatide-stimulated (10 µM, 20 µM) slice cultures (clear bar) were measured after 24 h of Olaparib exposure (black bar). Olaparib (100 nM) showed a significant decrease in **A** IL-6, **B** TNF-α, **C** IL-17A, and **D** CCL3, **E** MIF, and **F** IFN-γ release into the slice culture medium, as measured by ELISA. **A** Olaparib causes a significant reduction in IL-6 secretion from slice cultures when compared to the sulfatides group, **B** Olaparib also caused a significant decrease in TNF-α secretion from sulfatide-stimulated slice cultures. **C** Olaparib reduces IL-17A secretion, **D** CCL3, **E** MIF from sulfatide-stimulated slice cultures and **F** IFN-γ from sulfatide-stimulated slice cultures. Data from the ELISA assays are presented as mean ± SEM. IL-6, TNF-α, IL-17A, CCL3, MIF, and IFN-γ ELISA's were repeated n = 5. Statistical analysis was performed using a Two-way ANOVA followed by Tukey’s post hoc test; *p < 0.05, **p < 0.01, ***p < 0.001

PARP-1 has a key role in chronic inflammation in the context of many inflammatory-driven pathologies [20]. Given that our data show that Olaparib attenuates sulfatide-induced microglia activation, we investigated whether Olaparib can dampen sulfatide-induced inflammatory cytokine and chemokine release. Treatment of slice cultures with 100 nM Olaparib for 24 h, in the presence of sulfatides (10 µM and 20 µM), significantly decreased the sulfatide-induced release of IL-6 (Control: 8.8 vs. Olaparib: 4.4 pg/ml; 10 μM 182.4 vs. 21.6 pg/ml *p < 0.05; 20 μM 166.5 vs. 27.7 pg/ml *p < 0.05; Fig. 9A), TNF-α (Control 22.3 vs. Olaparib: 16.0 pg/ml; 10 μM 110.4 vs. 59.1 pg/ml *p < 0.05; 20 μM 118.9 vs. 70.2 pg/ml *p < 0.05; Fig. 9B), IL-17A (Control 61.5 vs. Olaparib: 37.2 pg/ml; 10 μM 769.4 vs. 93.0 pg/ml ***p < 0.001; 20 μM 762.2 vs. 82.2 pg/ml ***p < 0.001; Fig. 9C), CCL3 (Control 64.7 vs. Olaparib: 87.8 pg/ml; 10 μM 575.2 vs.442.5 pg/ml; 20 μM 629.3 vs. 423.2 pg/ml **p < 0.01; Fig. 9D), MIF (Control 869.2 vs. Olaparib: 885.0 pg/ml; 10 μM 2919.4 vs. 1695.4 pg/ml **p < 0.01; 20 μM 2317.0 vs. 1259.2 pg/ml *p < 0.05; Fig. 9E), and IFN-γ (Control 36.6 vs. Olaparib: 57.6 pg/ml; 10 μM 711.8 vs. 81 pg/ml ***p < 0.001; 20 μM 426.8 vs. 49.3 pg/ml ***p < 0.001; Fig. 9F). Taken together, these data suggest that sulfatide exposure promotes inflammation and that Olaparib can attenuate this by significantly reducing the secretion of pro-inflammatory cytokines and chemokines. This may in part explain the protective effects of PARP inhibition on sulfatide-induced demyelination.

### Positive Correlation Between Levels of Pro-Inflammatory Mediators and Demyelination

**Fig. 10:**
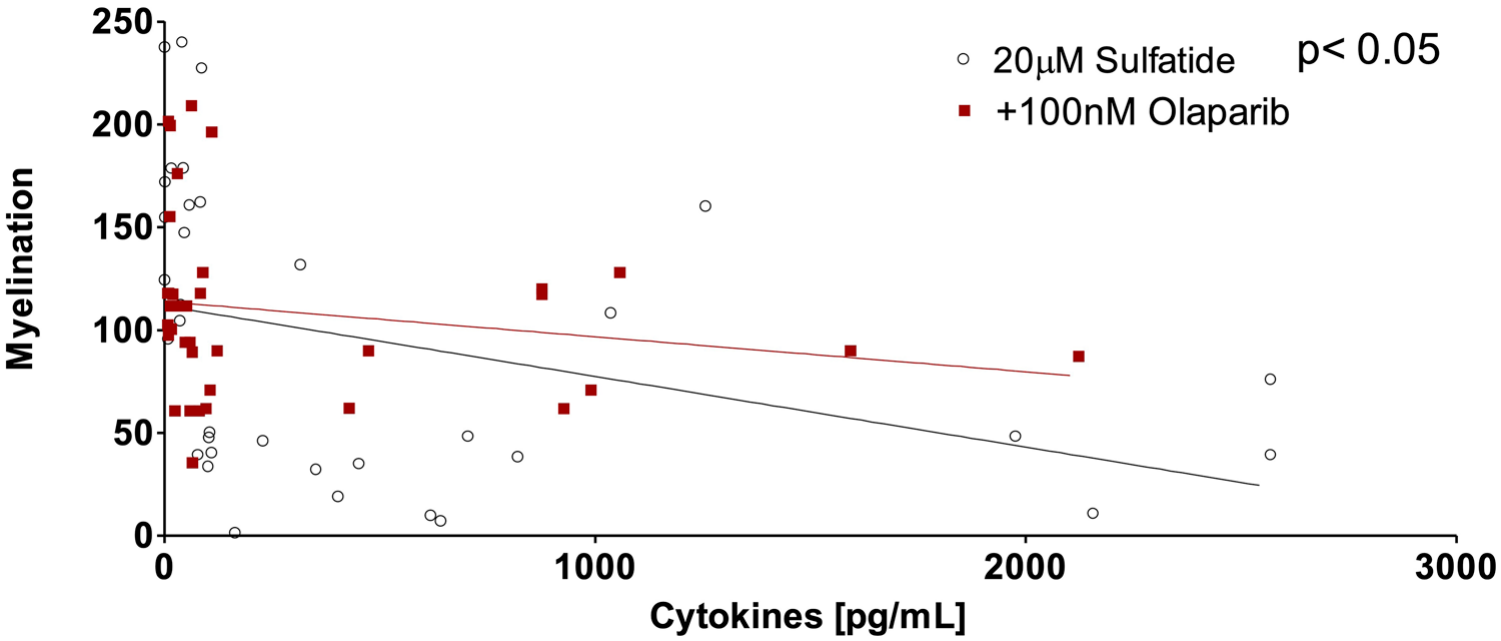
**Correlation between pro-inflammatory cytokine and chemokine release and demyelination in organotypic slice cultures.** Supernatants generated from the organotypic slice cultures treated with 20 µM sulfatide with or without 100 nM Olaparib and stained with MBP were used to perform ELISAs. The levels of the IL-6, IL-17A, TNF-α, CCL3, MIF, and IFN-γ were significantly positively correlated with demyelination induced by 20 µM sulfatide. Statistical analysis was performed using Correlation; *p < 0.05, n = 5

To further determine whether the elevated concentrations of cytokines and chemokines in sulfatide-treated slice cultures are associated with the severity of demyelination, a correlation analysis was conducted. Supernatants generated from organotypic slice cultures treated with 20 µM sulfatide with or without 100 nM Olaparib and stained with MBP were used to perform ELISAs. Interestingly, the overall secretion of IL-6, IL-17A, TNF-α, CCL3, MIF, and IFN-γ significantly correlated with a decrease in myelination measured by MBP staining (ρ = -0.3570, *p* = 0.0353) (Fig. 10). Treatment with Olaparib lessened this correlation by reducing both pro-inflammatory cytokine and chemokine secretion and attenuating demyelination (ρ = -0.1965, *p* = 0.2508) (Fig. 10). These data strongly indicate that sulfatide-induced demyelination is paralleled by pro-inflammatory cytokine and chemokine release in matched samples thus implicating sulfatide-induced inflammation as a key contributor to demyelination. Furthermore, these data indicate that Olaparib holds promise to simultaneously attenuate pro-inflammatory cytokine and chemokine release and demyelination in MLD.

### Olaparib Attenuates Sulfatide-Induced Levels of SMI-32

**Fig. 11:**
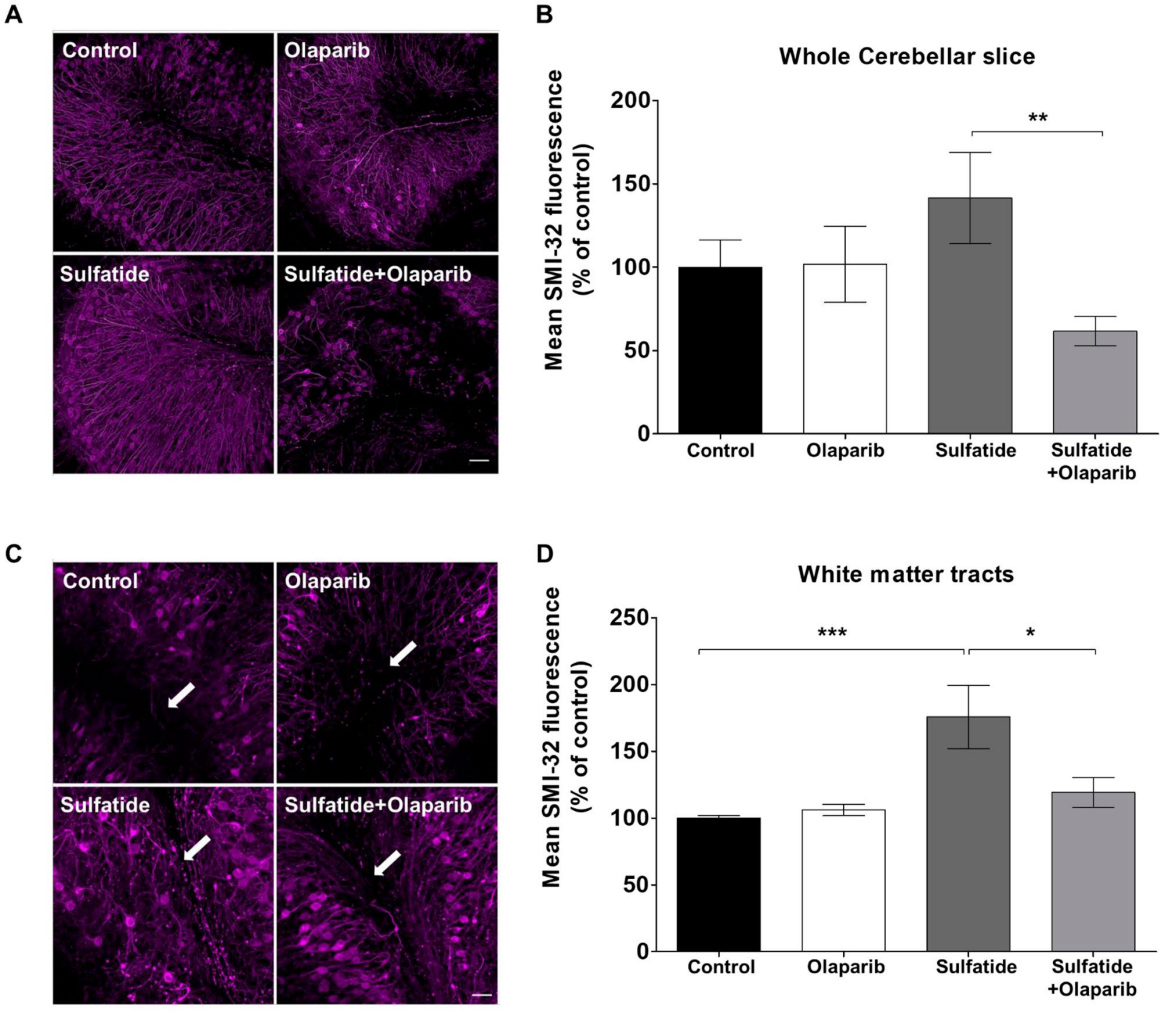
**Olaparib attenuates sulfatide-induced axonal damage.** OCS were treated with sulfatides (20 µM) in the presence or absence of Olaparib (100 nM) for 24 h. **A** Representative confocal images displaying SMI-32 immunostaining in whole cerebellar slice under treatment conditions indicated. Confocal images were captured at × 20 magnification. **B** Relative fluorescent intensity of SMI-32 staining in the whole cerebellar slice shows a protective effect of Olaparib on sulfatide-induced increase in SMI-32 fluorescence. **C** Representative confocal images displaying SMI-32 immunostaining in white matter tract under treatment conditions indicated. Confocal images were captured at × 20 magnification. **D** Relative fluorescent intensity of SMI-32 staining in major white matter tract regions shows a protective effect of Olaparib on sulfatide-induced increase in SMI-32 fluorescence. Data are expressed as a percentage of control and presented ± SEM compared with control (n = 5). Statistical significance was determined by One-way ANOVA followed by Tukey multiple comparison test, *p < 0.05, **p < 0.01, ***p < 0.001. A number of 100 images were analyzed per condition. Scale bar, 100 µm

SMI-32 is a non-phosphorylated epitope of NFH and a marker of axonal damage and demyelination [21–23]. Here, SMI-32 was used as a biomarker to examine the effects of Olaparib on sulfatide-induced axonal damage. Murine OCSs were treated with 20 μM sulfatide in the presence or absence of 100 nM Olaparib for 24 h and then fixed and stained for SMI-32. OCS treated with sulfatide displayed an increase in SMI-32 expression in the whole cerebellum (141.4% compared with control) (Fig. 11A and B) and in the axons of cerebellar Purkinje neurons (175.8%, ***p < 0.001 compared with control) (Fig. 11C and D). Importantly, treatment with Olaparib significantly attenuated the overall increase in SMI-32 fluorescence intensity in the cerebellum (Sulfatide: 141.4% vs Sulfatide + Olaparib: 61.5%, **p < 0.005) (Fig. 11A and B) and in the white matter tract (Sulfatide: 175.8% vs Sulfatide + Olaparib: 119.2%, *p < 0.05) (Fig. 11C and D). These results show for the first time that sulfatide accumulation increases SMI-32 staining within the axons of the major white matter tracts in the cerebellum, indicating axonal damage. Importantly, this axonal damage is attenuated by treatment with Olaparib.

## Discussion

**Fig. 12:**
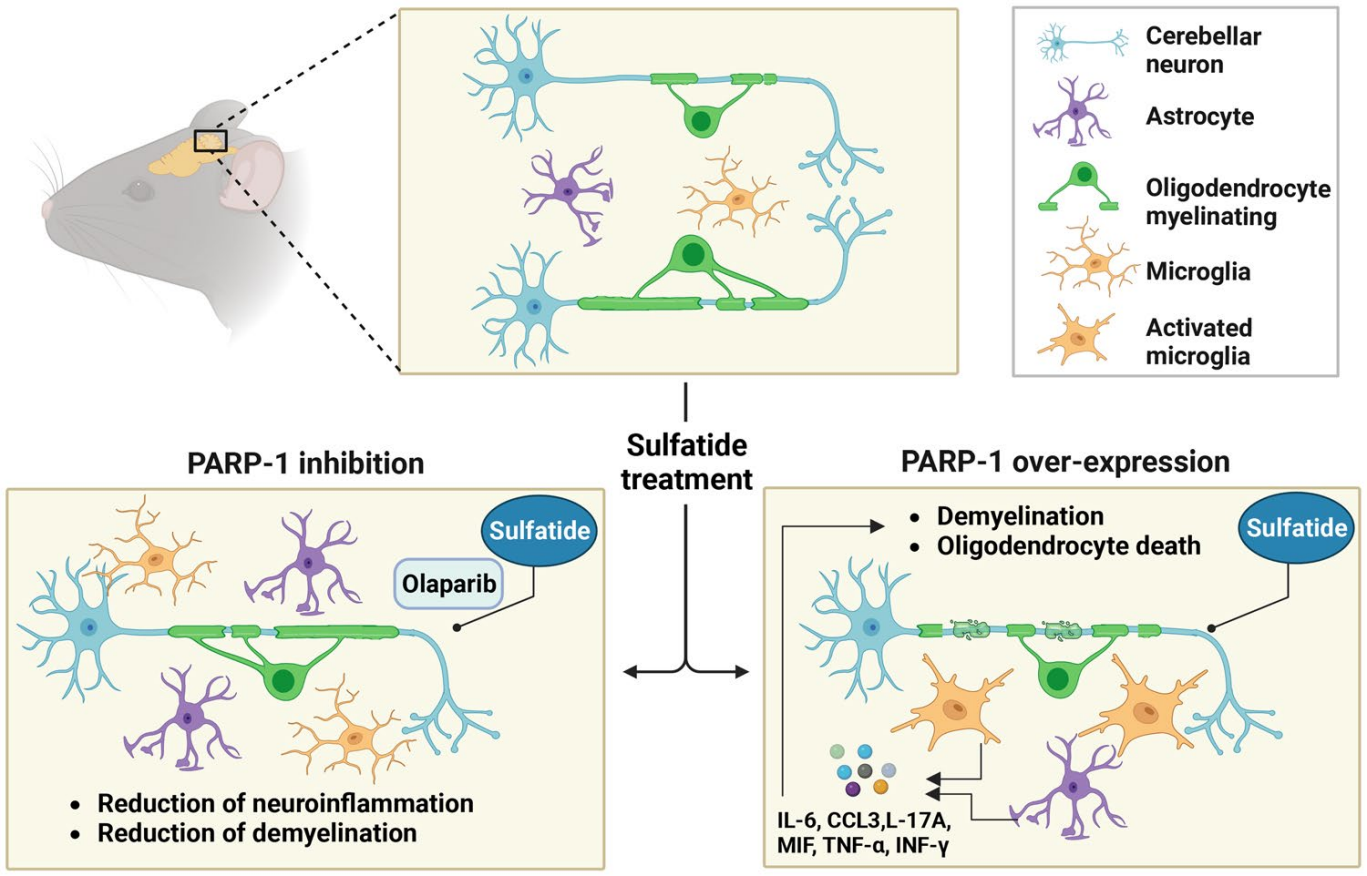
Proposed mechanism of action of Olaparib in sulfatide-treated mouse organotypic cerebellar slice cultures

In this current study, we examined the effect of sulfatide on markers of myelination in an intact mouse organotypic cerebellar slice culture model. We report for the first time that direct application of sulfatides induces demyelination of organotypic cerebellar slices in a concentration-dependent manner. Moreover, sulfatide-treated organotypic slice cultures appear to impair oligodendrocyte viability and axonal morphology. Olaparib showed strong efficacy in rescuing sulfatide-induced demyelination and rescued axonal degeneration caused by treatment with sulfatides. For the first time, our data show that sulfatides induced PARP-1 activation in mouse organotypic slice cultures, which was significantly attenuated by Olaparib. Sulfatides increased the secretion of pro-inflammatory cytokines and chemokines IL-6, IL-17A, TNF-α, CCL3, MIF, and IFN-γ by slice cultures, which could be suppressed by Olaparib. Lastly, the data showed that Olaparib dampened the augmented microglial proliferation/activation and astrocyte impairment in slice cultures treated with sulfatides. Taken together, this research suggests that PARP-1 plays a key role in sulfatide-induced inflammation and demyelination in mouse organotypic slice culture, which can be reversed by Olaparib (Fig. 12).

### Role of Sulfatide in Demyelination

Chronic demyelination and almost complete loss of oligodendrocytes are two of the major pathological features of MLD [4]. The hypothesis that supraphysiological levels of sulfatides kill oligodendrocytes and result in widespread demyelination is now widely accepted [24, 25]. Sulfatide levels in CSF and the sural nerve have been correlated to the severity of neuropathy in patients with MLD [25], and in a recent publication, increased levels of CSF sulfatide correlated with worse motor function in MLD [26]. The mechanisms by which sulfatides induce demyelination in MLD remain unclear at present. Increasing evidence suggest that the widespread demyelination and loss of oligodendrocytes observed in MLD could be due to sulfatide-induced chronic inflammation underpinned by excessive cytokine and chemokine release [3, 16].

Here, we examined the effect of sulfatide on markers of myelination in an intact cell culture model such as organotypic slice cultures. We report that the direct application of sulfatides induces demyelination of organotypic slice cultures in a concentration-dependent manner. These data corroborate the idea that sulfatides may directly induce demyelination in the brains of MLD patients. In addition, sulfatide-treated organotypic slice cultures show a significant oligodendrocyte death, rescued by Olaparib. Interestingly, slice cultures treated with Olaparib alone show oligodendrocyte loss suggesting that PARP-1’s enzymatic activity is necessary for oligodendrocyte development in physiological conditions.

Lastly, we demonstrated that PARP-1 inhibition reduces sulfatide-induced axonal damage in mouse slice cultures. This is in agreement with data from pre-clinical model of MLD showing that elevated sulfatide levels in neurons are accompanied by a significant axonal degeneration.

### Role of Sulfatide in Neuroinflammation

Sulfatides represent an important, relatively unexplored component of immune regulation in the CNS [27]. MLD patients exhibit augmented levels of pro-inflammatory cytokines and chemokines including CCL2, IL-1Ra, IL-8, and CCL4 in the CSF, and sulfatides are known to influence a variety of immune cells, such as neutrophils, dendritic cells, B cells, and microglia in the CNS [27–29]. Data presented here demonstrate that sulfatide-treated organotypic slice cultures exhibit an enhanced inflammatory profile characterized by augmented microglial proliferation/activation, astrocyte impairment, and elevated levels of pro-inflammatory cytokines and chemokines such as IL-6, IL-17A, TNF-α, CCL3, MIF, and IFN-γ. Activation of pro-inflammatory pathways in the brain, including IL-6 pathway, TNF-α, IL-17A, and MIF comprises a potential point of convergence between chronic neuroinflammation and neurodegeneration in many brain diseases such as AD and MS, so it is rational to hypothesize that the elevation of these cytokines contributes to demyelination in MLD [30–32]. Moreover, the elevated levels of CCL3, IFN-γ, and IL-6 observed here is in line with elevated levels observed in the preclinical stages of MLD [3]. Nowadays it is generally accepted that these cytokines promote pathology by activating glial cells and by recruiting peripheral immune cells such as granulocytes and T cells [3, 33]. CCL3, IFN-γ, and TNF-α are T helper 1 cytokines and they play a pathogenic role in other demyelinating diseases namely MS in which they drive the recruitment and activation of immune cells and elicit toxic or proapoptotic effects on oligodendrocytes [33]. The production of IL-17A has been demonstrated as pivotal for autoimmune demyelination and it appears to be almost exclusively T helper 17 cytokine induced by elevated levels of IL-6 [34]. Our findings together with data in the literature, strengthen the idea that neuroinflammation could be a major driver of MLD, therefore enhanced levels of myelination and less axonal degeneration could be achieved via modulation of immune pathways, microglia, and astrocytes.

### Identifying the Role of PARP-1 and the Therapeutic Utility of Olaparib in MLD

PARP-1 is expressed by all brain cells in the central nervous system (CNS) and is involved in the synthesis of PAR, cell differentiation and maturation, regulation of cholinergic and glutamatergic signaling, and memory formation [10, 35, 36]. Previous studies have reported that PARP-1 chronic activation, which occurs in many demyelinating disorders, causes neuronal death, axonal degeneration, and impairment of essential myelin proteins (e.g., myelin oligodendrocyte glycoprotein (MOG), myelin basic protein (MBP), myelin-associated glycoprotein (MAG)) [14]. Furthermore, PAR, the marker of PARP-1 activation, accumulates in astrocytes as well as oligodendrocytes, microglia, and neurons, surrounding demyelinated plaques [7]. Our data show that sulfatide-treated slice cultures express higher levels of PARP-1, suggesting that PARP-1 may contribute to demyelination and neuroinflammation in MLD.

Given that PARP-1 inhibition with selective inhibitors lessens neurodegeneration and demyelination in several animal disease models (e.g., PD, AD, and MS) and improves symptoms, via a reduction in the expression of inflammatory cytokines, we investigated the effects of Olaparib, a PARP inhibitor, on our sulfatide-treated slice culture model.

Olaparib proved to reduce PARP-1 expression and prevent sulfatide-induced demyelination. It is not yet known if Olaparib exerts a direct beneficial effect on oligodendrocytes or an indirect protective mechanism of action on myelinated axons. Indeed, both scenarios are possible. Importantly, the inflammatory response associated with demyelination was deeply reduced by Olaparib as shown by the rescue in astrocyte loss, the decrease in proliferation and activation of microglial Iba1, as well as pro-inflammatory molecules levels. Taken together, these reports suggest that drugs inhibiting PARP-1 activity protect from neuroinflammation in MLD model via immunomodulation and direct neuroprotection and provide strong evidence for the utility of PARP-1 inhibitors in MLD and neuroinflammation. Further insights into the function of PARP-1 in brain homeostasis and dysregulation in pathological states may assist in the development of PARP-1 inhibitors for MLD.

## Conclusion

Here we demonstrated that sulfatide treatment induces demyelination, PARP-1 expression, axonal damage, astrocyte impairment, and elevates pro-inflammatory cytokine and chemokine levels in murine organotypic slice culture. Our data also indicate that sulfatides increase microglial proliferation and promote an ameboid morphology. Importantly, all the deleterious effects of sulfatides were attenuated by the PARP-1 inhibitor Olaparib. Overall, our study data suggest that PARP-1 inhibition may be a therapeutic option in MLD, where marketed drugs such as Olaparib may be worthy of further investigation (Fig. 12).

## Data Availability

The data that support the findings of this study are available from the corresponding author upon reasonable request.
